# Supplementary material for: Stress-Responsive Gene Expression, Metabolic, Physiological, and Agronomic Responses by Consortium Nano-Silica with Trichoderma against Drought Stress in Bread Wheat
Source: Int J Mol Sci. 2024 Oct 11;25(20):10954. doi: 10.3390/ijms252010954 (PMC11507820; doi:10.3390/ijms252010954)
Supplement: Supplementary file 1 [file ijms-25-10954-s001.zip › ijms-3197139-supplementary.pdf]

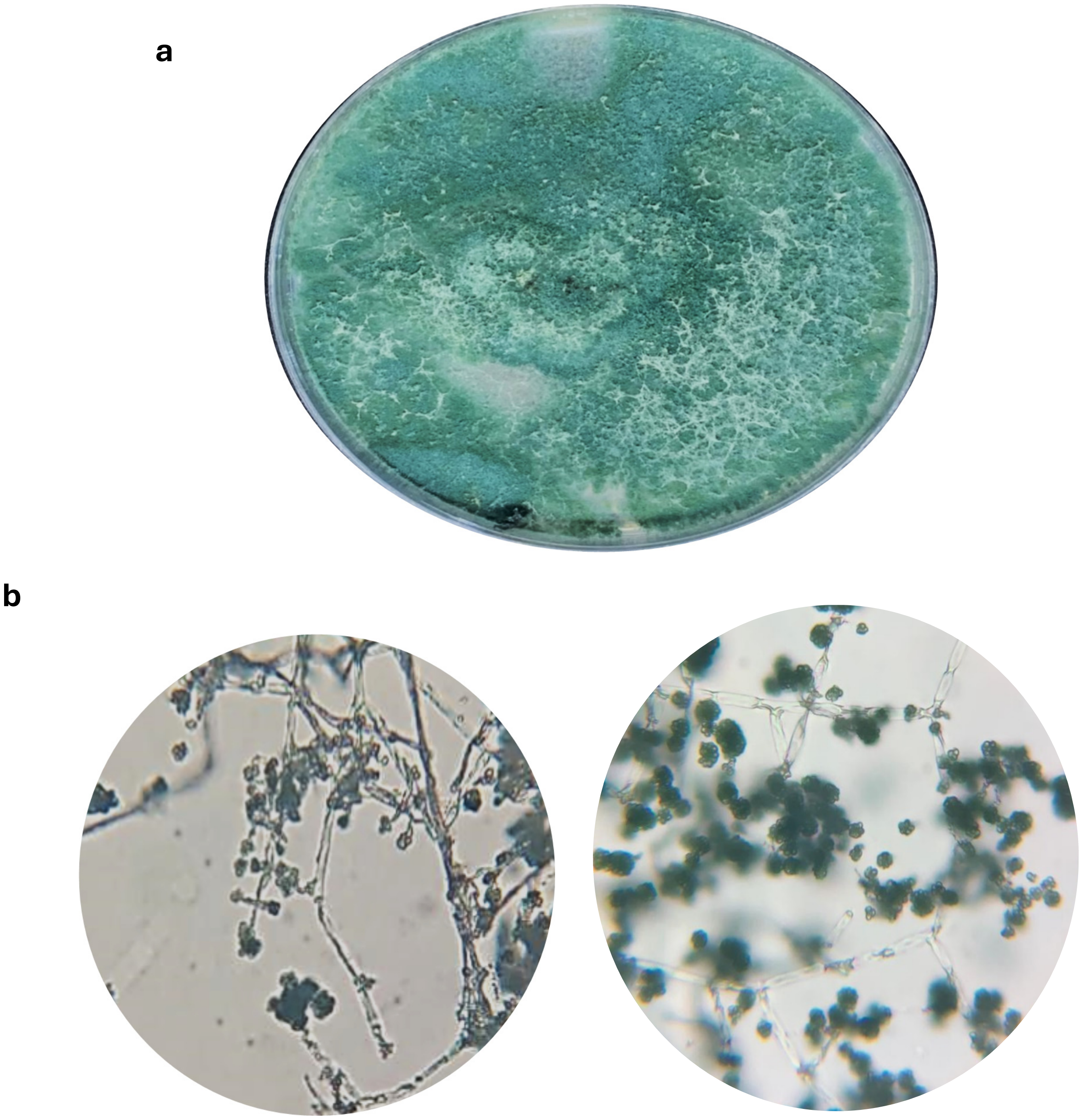

Supplementary Figure S1: Cultural and morphological characterization of *Trichoderma harzianum.* a) Culture and macroscopic colonies, and b) Microscopic identification at 100x.

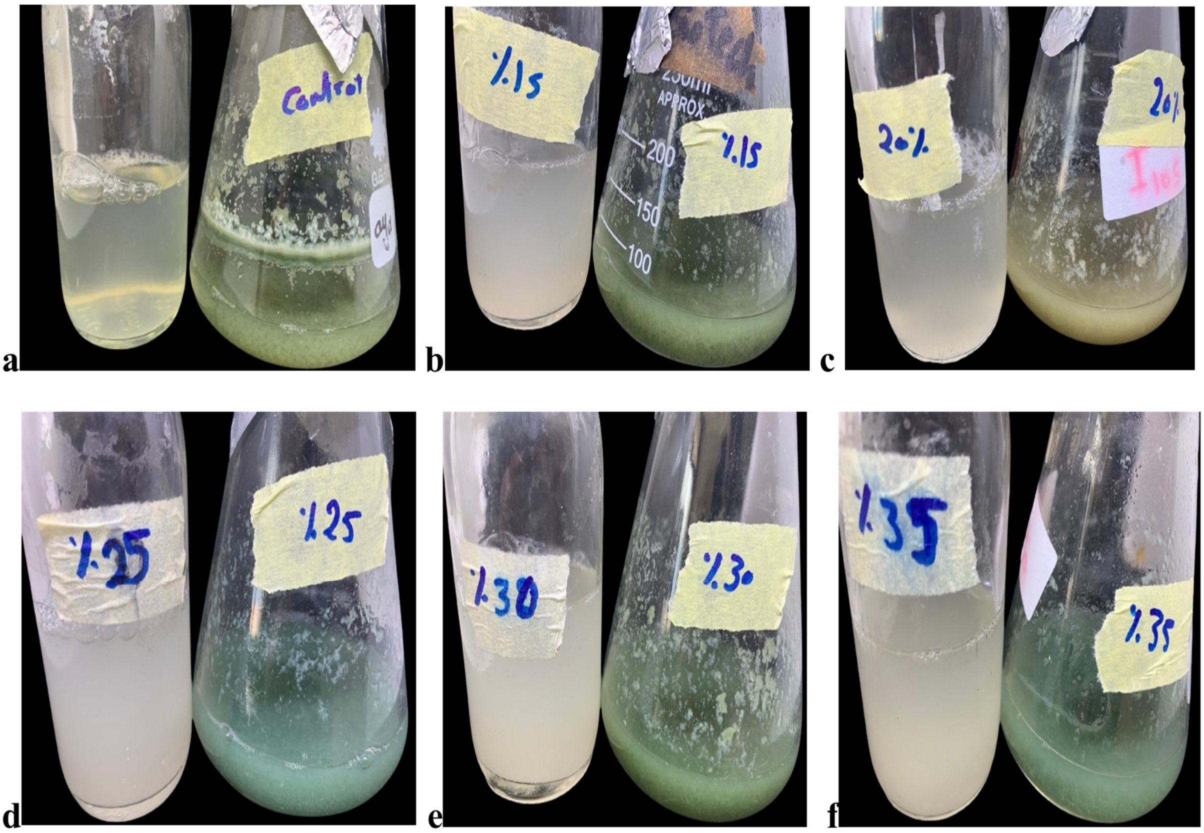

Supplementary Figure S2. Estimation of *T. harzianum* in different concentrations of Polyethylene glycol (PEG) a) 0%; b) 15%; c) 20%; d) 25%; e) 30% and f) 35%.

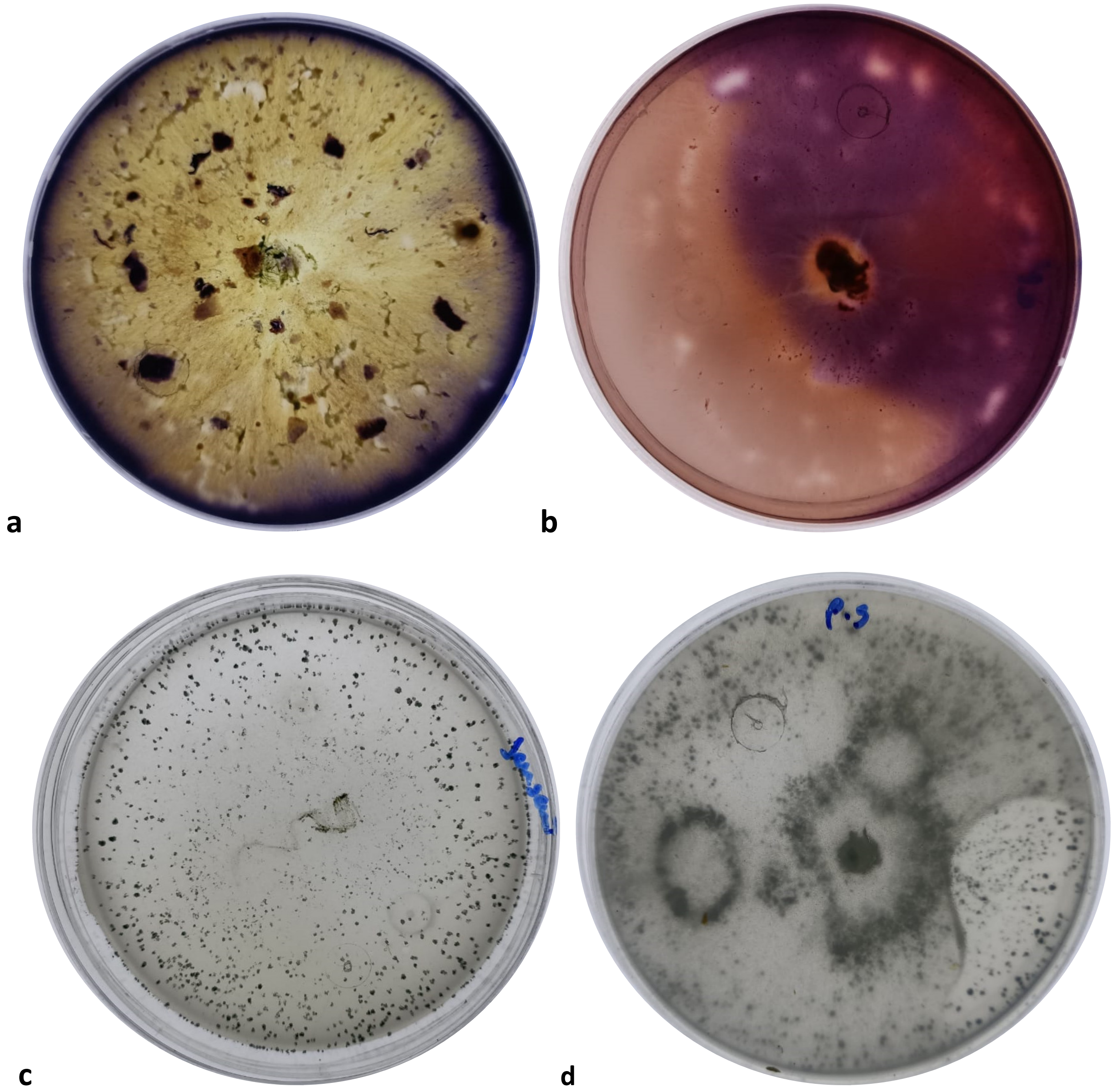

Supplementary Figure S3. Screening for *T. harzianum* plant growth promoting criteria: a) Amylase; b) Cellulase; c) Nitrogen fixation and d) Phosphate solubilization.

Supplementary Table S1. XRD analysis of silica nanoparticles.

2 Theta	d Value (A)	Intensity%	hkl	Size (nm)
22.103	4.018	12.6	100	79.12
27.55	3.23	28	110	51.92
31.846	2.80	100	110	67.86
45.582	1.98	65	200	70.19
54.026	1.69	13	211	73.87
56.637	1.62	28.2	211	66.32
66.324	1.40	12.9	221	54.25
75.392	1.25	23.8	311	80.44

Hkl, Miller indices
